# Aging of the Hematopoietic System: Mechanisms, Consequences, and Systemic Interactions

**DOI:** 10.1111/acel.70385

**Published:** 2026-01-16

**Authors:** Masashi Miyawaki, Seiji Hashimoto, Sumito Ogawa, Yoshitaka Kase

**Affiliations:** ^1^ Department of Geriatric Medicine, Graduate School of Medicine The University of Tokyo Tokyo Japan; ^2^ Division of Geroscience, International Center for Brain Science (ICBS) Fujita Health University Toyoake‐shi Aichi Japan

**Keywords:** aging, healthspan, hematopoiesis, hematopoietic stem cells, inflammation

## Abstract

The aging of the hematopoietic system is central to physiological aging, with profound consequences for immune competence, tissue regeneration, and systemic health. Age‐related changes manifest as altered blood cell composition, functional decline in hematopoietic stem cells (HSCs), and deterioration of the bone marrow niche. Beyond hematologic dysfunction, hematopoietic aging acts as a systemic amplifier of age‐related diseases through clonal hematopoiesis and inflammatory remodeling. This review integrates recent insights into the mechanisms and systemic impacts of hematopoietic aging, reframing it as a modifiable axis of systemic aging. We highlight emerging rejuvenation strategies—senolytics, metabolic reprogramming, and microbiota‐targeted therapies—that aim to restore hematopoietic and immune function, offering promising avenues to improve healthspan and reduce age‐related multimorbidity.

## Graphical abstract

Hematopoietic aging arises from intrinsic stem‐cell decline and bone‐marrow niche dysfunction, leading to myeloid skewing, immunosenescence, and clonal hematopoiesis. These changes drive systemic inflammation and multi‐organ disease, which in turn further disrupt the bone‐marrow niche and hematopoietic stem cell (HSC) function, creating a vicious cycle that accelerates organismal aging.

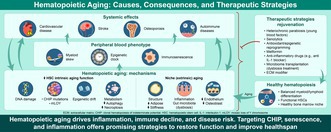

## 1 Introduction

Hematopoiesis is a lifelong process in which the body maintains the production of blood and immune cells from a reservoir of hematopoietic stem cells (HSCs) (Pinho and Frenette 2019). This dynamic system maintains homeostasis and ensures immune surveillance, tissue repair, and oxygen transport throughout life. However, aging imposes significant challenges on the hematopoietic system, resulting in functional impairments and increased susceptibility to illness.

The aging hematopoietic system is characterized by several hallmark features: myeloid‐biased differentiation, reduced lymphopoiesis, diminished regenerative capacity, and increased incidence of clonal hematopoiesis (Pang et al. 2011; De Haan and Lazare 2018; Jaiswal et al. 2014; Evans and Walsh 2023). These changes result from a complex interplay of intrinsic HSC alterations—including DNA damage, epigenetic drift, and metabolic dysfunction—and extrinsic factors, such as niche aging and systemic inflammation. While systemic inflammation is often regarded as a hallmark of aging (“inflammaging”), recent evidence suggests that its extent may be amplified by industrialized lifestyles rather than being an inevitable consequence of aging itself (Franck et al. 2025). This perspective underscores that some extrinsic drivers of hematopoietic aging may be environmentally modifiable.

Clinically, hematopoietic aging contributes to immunosenescence, increased vulnerability to infections, poor responses to vaccines, and a higher risk of hematologic malignancies (Liu et al. 2023). Notably, the discovery of clonal hematopoiesis with indeterminate potential (CHIP) has established direct links between aging hematopoietic clones and systemic diseases, particularly cardiovascular disease (Jaiswal et al. 2017; Evans and Walsh 2023). These findings highlight that hematopoietic aging is not confined to the hematologic domain but exerts widespread effects on organismal health.

This review comprehensively compiles the available data about the molecular mechanisms driving hematopoietic aging and reframes the aging hematopoietic system as a modifiable axis of organismal aging. We highlight emerging evidence that hematopoietic aging reflects and amplifies systemic pathology through chronic inflammation, clonal expansion, and inter‐organ communication. Furthermore, we discuss promising rejuvenation strategies—including senolytics, metabolic reprogramming, and microbiota‐targeted therapies—that aim to restore hematopoietic and immune function, offering novel avenues to extend healthspan and counteract age‐related multimorbidity.

## 2 Age‐Related Changes in the Peripheral Blood of the Elderly

### 2.1 Myeloid Skewing

In elderly humans, peripheral blood often shows relative myeloid predominance and reduced lymphocyte counts (Pang et al. 2011; Pellegrino et al. 2024).

Recent lineage‐tracing studies of aged mice showed that HSC‐derived differentiation declines across all lineages with only partial compensation in the myeloid compartment (Säwen et al. 2018; Jang et al. 2023; Konturek‐Ciesla et al. 2023). Consistently, in situ barcoding of physiologically aged mice demonstrated that the increased abundance of myeloid cells during aging results from a higher number of HSPC clones contributing to myelopoiesis, rather than enhanced myeloid output per clone, refining the interpretation of “myeloid bias” from intrinsic lineage skewing to a clonal compensation mechanism (Urbanus et al. 2023).

Consistently in humans, recent single‐cell analyses of healthy bone marrow revealed that myeloid shift is not driven by an expansion of myeloid progenitors but rather by a decline in overall differentiation activity and a disproportionate loss of lymphoid potential (Komic et al. 2025). Finally, high‐resolution clonal tracking across both mice and humans has demonstrated that clonal expansions—rather than uniform lineage shifts—account for much of the apparent myeloid bias (Scherer et al. 2025). Together, these findings redefine age‐associated myeloid skewing as an emergent property of impaired differentiation and clonal imbalance, rather than a simple shift in lineage priming.

Alterations in HSPC composition and clonal architecture during aging may also underlie the dysregulated megakaryopoiesis and platelet function that accompany myeloid skewing (Reusswig et al. 2024). Single‐cell transcriptomic analyses in mice have shown that aging increases the proportion of HSCs committing to the megakaryocyte–erythroid differentiation trajectory (Hérault et al. 2021). Interestingly, studies in mice have shown that aged HSCs can exhibit a direct differentiation pathway toward megakaryocyte progenitors, bypassing canonical progenitor stages (Poscablo et al. 2024). Extending this paradigm, a recent study identified a rare population of HSC‐derived noncanonical megakaryocyte progenitors that expands with age and exhibits enhanced platelet‐generating capacity, directly linking HSC heterogeneity to age‐related thrombopoiesis (Manso et al. 2025). Complementing these mouse studies, recent single‐cell and in vivo barcoding analyses in humans have demonstrated that platelet‐biased HSCs increase in frequency and transcriptional priming with age, directly linking HSC heterogeneity to age‐associated thrombopoiesis in the human hematopoietic system (Aksöz et al. 2024). At the clinical level, aging is associated with changes in platelet reactivity, turnover, and activation profiles, potentially fostering a pro‐thrombotic, pro‐inflammatory state (Martin et al. 2006; White 2003).

### 2.2 Immunosenescence and Its Manifestations

In humans, immunosenescence denotes a progressive reduction in adaptive immune competence with age. Thymic involution reduces naive T‐cell output, resulting in diminished T‐cell receptor (TCR) diversity (Nikolich‐Žugich 2018; Liu et al. 2023). The peripheral T cells shift toward memory and senescent phenotypes, particularly CD8^+^ T cells, which accumulate DNA damage and exhibit impaired function. B‐cell output and diversity decline with reduced class‐switch recombination and somatic hypermutation. Consequently, elderly individuals experience poor responses to vaccination, increased infection susceptibility, and elevated cancer risk.

A specific subset of T cells termed senescence‐associated T (SA‐T) cells has also been implicated as a key contributor to chronic inflammation and age‐associated diseases (Shimatani et al. 2009; Fukushima et al. 2018). SA‐T cells were first described in mice and are characterized by the loss of co‐stimulatory molecules like CD28, high expression of inhibitory receptors, and release of pro‐inflammatory cytokines such as osteopontin (Tahir et al. 2015). With advancing age, these cells accumulate and reside in inflamed tissues, promoting the inflammaging phenotype. Consistent with this, in humans, expansions of highly differentiated/senescent T cell subsets are associated with metabolic and autoimmune pathology (Robertson and Hansson 2006; Lee et al. 2019; Lu et al. 2023). Their pro‐inflammatory activity exacerbates tissue damage, sustains chronic inflammation, and represents a potential therapeutic target for mitigating immunosenescence‐related pathology and improving immune resilience in aging populations.

Moreover, in mice, recent studies have suggested that bacteria‐induced B cell senescence contributes to immune decline with age. Persistent stimulation by commensal gut bacteria such as *Bacteroides acidifaciens* induces senescence in germinal center (GC) B cells, particularly in Peyer's patches and isolated lymphoid follicles (Kawamoto et al. 2023). This leads to reduced production and diversity of IgA, impairing mucosal immunity and altering the gut microbiota composition, thereby creating a feedback loop of inflammation and immune dysregulation.

Supercentenarians, individuals aged 110 years or older, provide further unique insights into successful hematopoietic aging (Hashimoto et al. 2019). Despite their extreme age, supercentenarians exhibit remarkable immune competence. Studies have shown expanded cytotoxic CD4^+^ T‐cell populations and reduced clonal hematopoiesis compared to younger individuals (Hashimoto et al. 2019).

Beyond the adaptive arm, innate compartments also age. In mice, p16+ senescent cells including alveolar macrophages upregulate PD‐L1, enabling immune evasion and sustaining inflammation (Majewska et al. 2024). In mice and humans, senescent cells broadly upregulate the ganglioside GD3, which suppresses NK cell–mediated clearance via Siglec interactions; blockade of GD3 restores NK cytotoxicity and mitigates age‐related tissue fibrosis in vivo (Iltis et al. 2025).

These age‐associated changes in peripheral blood reflect the broader remodeling of the hematopoietic system and establish the foundation for understanding downstream impacts on immunity, disease risk, and potential rejuvenation strategies.

## 3 Intrinsic Aging of Hematopoietic Stem Cells

### 3.1 Impaired Self‐Renewal, Repopulating Capacity, and Differentiation Bias in Aging HSCs

Hematopoietic stem cells (HSCs) exhibit progressive functional decline with age and are marked by reduced self‐renewal, diminished engraftment potential, and lineage skewing. Although the count of phenotypically defined HSCs increases with age, their quality and regenerative capacity deteriorate (De Haan and Lazare 2018; Sudo et al. 2000; Ogawa et al. 2000; Beerman et al. 2010). Aged HSCs exhibit reduced capacity to remain in a quiescent, non‐cycling state, leading to exhaustion and hematopoietic dysfunction, which is supported by the fact that increasing donor age is associated with worse outcome in HSC transplantation (Pruitt et al. 2023).

An integrative meta‐analysis of murine HSC transcriptomes defined an aging signature enriched for membrane molecules such as P‐selectin and skewed toward gene upregulation, consistent with a transcriptionally activated state in aged HSCs supported by increased RNA polymeraseII activity and chromatin accessibility (Flohr Svendsen et al. 2021).

Transcriptomic analyses in both mice and humans demonstrate age‐associated reduction in lymphoid gene programs and increased myeloid output, consistent with functional skewing (Beerman et al. 2010; Kowalczyk et al. 2015). However, accumulating evidence suggests that these alterations do not simply reflect a binary deregulation of lymphoid versus myeloid differentiation programs at the transcriptional level. Instead, single‐cell analyses of murine HSC indicate that aging is prominently associated with defects in cell cycle regulation, including altered G1 phase dynamics and proliferation‐driven functional decline, which secondarily influence lineage output (Kowalczyk et al. 2015; Kirschner et al. 2017). Notably, recent analyses of human HSCs demonstrate a progressive, intrinsic impairment in mitogenic responsiveness and cell cycle entry with advancing age, characterized by delayed G1 progression and reduced AKT signaling, providing direct evidence that dysregulated cell cycle control is a conserved hallmark of intrinsic HSC aging across species (Hammond et al. 2023). Florian et al. further showed that disruption of HSC polarity through Cdc42 dysregulation impairs self‐renewal, highlighting cytoskeletal changes as a causal mechanism (Florian et al. 2018). More recently, Su et al. identified CD49b‐defined subsets with differential vulnerability to aging: myeloid‐biased CD49b^−^ HSCs expand and dominate with age, while lymphoid‐biased CD49b^+^ HSCs decline (Su et al. 2024). Notably, CD49b^−^ HSCs retain relatively preserved self‐renewal capacity even in aged mice, yet their differentiation output remains predominantly myeloid‐biased, indicating that preservation of stemness does not equate to maintenance of balanced lineage potential. Together, these findings from murine and human studies indicate that intrinsic functional decline and shifts in HSC subset composition converge to impair hematopoiesis with aging.

### 3.2 DNA Damage Accumulation and Genomic Instability

One major driver of intrinsic HSC aging is the accumulation of DNA damage from replication stress, oxidative injury, and declining repair capacity. In mice, aged or DNA‐repair–deficient HSCs accumulate damage and lose function, and a p53‐dependent differentiation checkpoint limits self‐renewal after genotoxic stress (Rossi et al. 2007; Wang et al. 2012). Replication stress has been shown to be a potent cause of functional decline in murine aging HSCs (Flach et al. 2014).

Mitochondrial ROS and impaired quality control further exacerbate genomic instability. Mouse studies demonstrate that FOXO3A‐driven autophagy protects HSCs from stress, and its disruption compromises HSC maintenance (Bratic and Larsson 2013; Warr et al. 2013).

In humans, accumulation of DNA damage with age is detectable in CD34^+^ HSPCs (γH2AX foci) from older individuals (Rübe et al. 2011). Moreover, human HSCs subjected to repeated proliferation (serial transplantation/xenotransplant settings) develop progressive ROS‐mediated oxidative DNA damage with activation of ATM–CHK2–FOXO3a pathways, leading to cell‐cycle arrest and senescence‐like changes; antioxidant treatment with N‐acetylcysteine mitigated this damage and preserved function (Yahata et al. 2011).

### 3.3 Clonal Hematopoiesis: Somatic Mutation and Chromosomal Mosaicism

Clonal hematopoiesis of indeterminate potential (CHIP) arises from somatic mutations in genes such as *DNMT3A*, *TET2*, and *ASXL1*, which confer a competitive advantage to mutant HSC clones. CHIP prevalence rises with advancing age and correlates with elevated risks of hematologic malignancies, cardiovascular disease, and overall mortality (Jaiswal et al. 2017; Evans and Walsh 2023). Large cohort studies have demonstrated that CHIP is detectable in over 10%–20% of individuals over 70 years (Jaiswal et al. 2014; Young et al. 2016; Watson et al. 2020).

CHIP exemplifies how age‐related intrinsic changes in HSCs contribute to systemic pathologies. Recent studies have suggested that CHIP‐associated mutations alter immune cell function, driving chronic inflammation and age‐related disease, as will be examined more fully in a dedicated section below.

In addition to single nucleotide mutations, chromosomal abnormalities contribute significantly to clonal hematopoiesis. Mosaic chromosomal alterations (mCAs), including copy number gains, losses, and loss of heterozygosity, as well as mosaic loss of the Y chromosome (mLOY), are key components of somatic mosaicism in hematopoietic cells. These abnormalities increase in prevalence with age and represent an alternative driver of clonal expansion distinct from classical gene mutations (Forsberg et al. 2014; Bruhn‐Olszewska et al. 2025). By the age of 70, up to 40% of men may exhibit mLOY in their hematopoietic cells. Importantly, individuals with mCAs exhibit greater susceptibility to hematologic malignancies and cardiovascular events, similar to CHIP (Forsberg et al. 2014; Bruhn‐Olszewska et al. 2025; Thompson et al. 2019; Sano and Walsh 2025). mLOY, in particular, is considered the most frequent somatic mosaicism observed in blood cells and serves as a biomarker for genomic instability and age‐related disease.

Recent high‐resolution genomic analyses have further advanced our understanding of age‐related clonal hematopoiesis. A pivotal study utilizing whole‐genome sequencing of over 3500 single‐cell–derived hematopoietic colonies revealed that although hematopoiesis remains polyclonal up to around 65 years of age, a marked reduction in clonal diversity occurs thereafter (Mitchell et al. 2022). In individuals > 70 years of age, 30%–60% of hematopoiesis cases were driven by only 12–18 dominant clones. These clones typically began expanding decades earlier, often before age 40, and the majority lacked known driver mutations. This suggests that the accumulation of moderate‐effect mutations under lifelong positive selection gradually primes the hematopoietic system for an abrupt shift toward oligoclonality in late life.

### 3.4 Epigenetic Drift and Transcriptional Dysregulation

Aging induces widespread epigenetic alterations in HSCs. Genome‐wide DNA‐methylation studies show that aging is accompanied by hypermethylation and repression of lymphoid‐associated genes, whereas a substantial subset of HSC identity‐defining genes–many of which are linked to myeloid‐biased output–exhibit relative hypomethylation with increased expression, consistent with lineage skewing (Beerman and Rossi 2015; Sun et al. 2014). Chromatin remodeling defects, loss of histone modification, and altered non‐coding RNA expression further intensify HSC dysfunction (Sun et al. 2014; Itokawa et al. 2022). In parallel, aged human HSCs undergo extensive epigenetic reprogramming of enhancers and promoters that mirror leukemic transformation.

Notably, the genes most frequently mutated in CHIP—*DNMT3A*, *TET2*, *ASXL1*—are epigenetic regulators identified in human population studies (Jaiswal et al. 2014; Young et al. 2016; Watson et al. 2020). Mechanistic work in mice shows that DNMT3A and TET2 cooperate to maintain DNA‐methylation homeostasis; loss‐of‐function perturbs heterochromatin organization and dysregulates immune/retroelement programs, changes that impair HSC function and favor myeloid output (Hong et al. 2023).

Together, cross‐species data support a model in which epigenetic drift within HSCs—amplified by mutations in epigenetic regulators—contributes to age‐associated transcriptional bias and functional decline.

### 3.5 Metabolic and Stress‐Response Pathway in Aging HSCs

**FIGURE 1:**
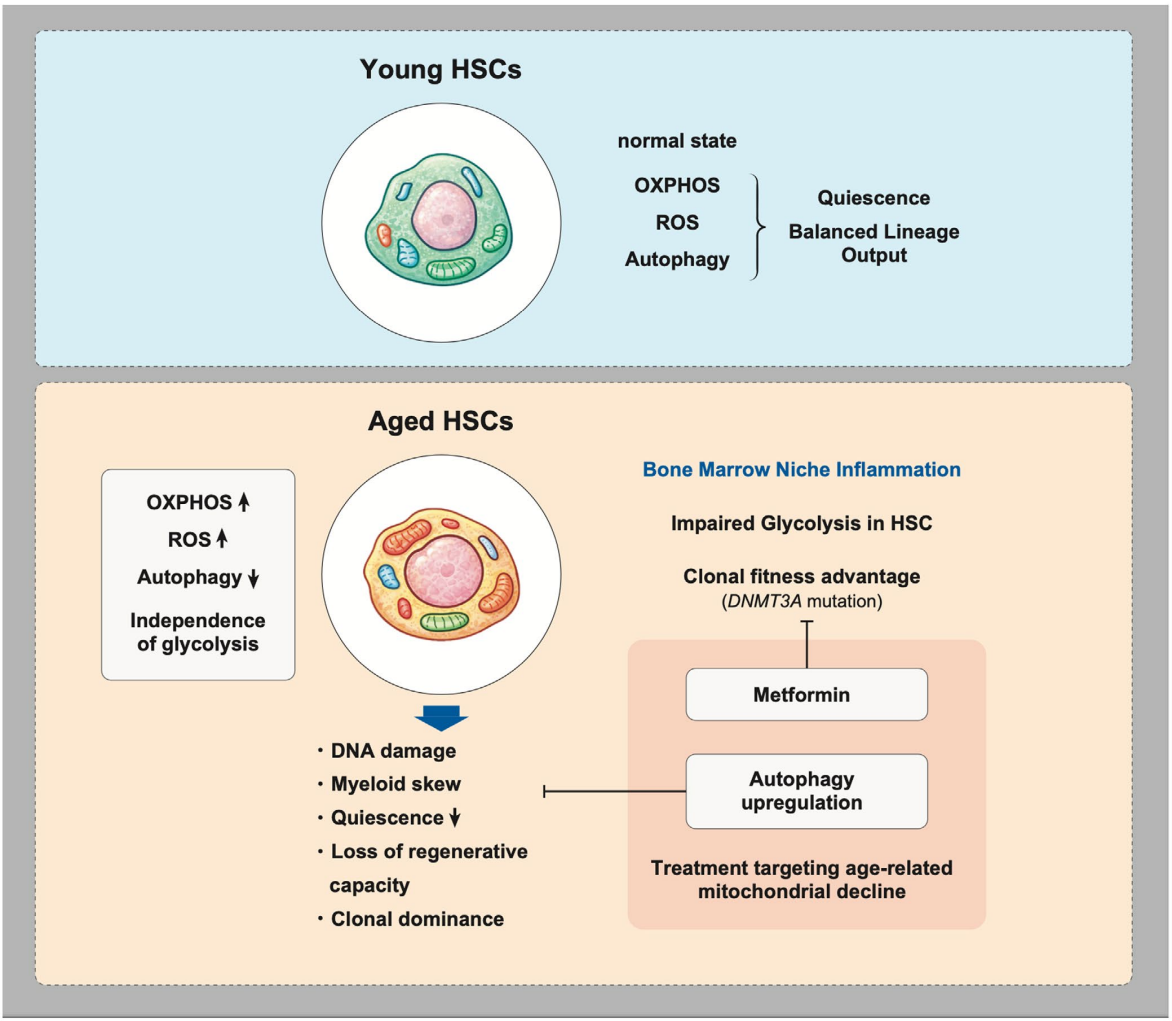
Mitochondrial and metabolic reprogramming in aged hematopoietic stem cells. Conceptual model illustrating mitochondrial and metabolic alterations accompanying hematopoietic stem cell aging. Young hematopoietic stem cells (HSCs) maintain quiescence and balanced lineage output through tightly regulated oxidative phosphorylation (OXPHOS), reactive oxygen species (ROS) levels, and autophagy. In contrast, aged HSCs exhibit increased reliance on OXPHOS, elevated ROS, and impaired quality‐control pathways, leading to DNA damage, myeloid‐biased differentiation, reduced quiescence, diminished regenerative capacity, and clonal dominance. Age‐associated inflammation in the bone marrow niche further disrupts glycolytic metabolism in HSCs, while adaptive changes in mitochondrial metabolism can confer a relative fitness advantage to specific mutant HSC clones (e.g., *DNMT3A*).

In addition to increased reliance on OXPHOS, recent studies have revealed that aged HSCs acquire complex metabolic adaptations involving mitochondrial pathways. Morganti and Ito reported that aged murine HSCs display increased mitochondrial oxidative phosphorylation and excessive ROS production, contributing to DNA damage, myeloid skewing, and loss of regenerative capacity (Morganti and Ito 2021) (Figure 1). Mitochondrial quality control mechanisms, including the FOXO3 and SIRT3 pathways, become impaired with age, intensifying oxidative stress and metabolic dysfunction.

Interestingly, Watanuki et al. demonstrated that aged HSCs gain metabolic resilience by upregulating mitochondrial ATP production via SDHAF1 accumulation (Watanuki et al. 2024). This adaptation allows aged HSCs to maintain energy production and survive independently of glycolysis, particularly under physiological low‐cytokine conditions, but may promote clonal dominance and functional decline.

Moreover, Dellorusso et al. found that persistent inflammation within the aged bone marrow niche impairs glycolysis in HSCs through SOCS3‐mediated AKT/FoxO inhibition in murine models (Dellorusso et al. 2024). In response, autophagy is activated as a protective mechanism to preserve HSC quiescence and function. Inducing autophagy transiently through fasting followed by refeeding rejuvenates glycolytic activity and enhances the regenerative capacity of aged HSCs, highlighting autophagy as a potential therapeutic target to reverse age‐related hematopoietic decline.

Interestingly, a new report highlighted that a subpopulation of aged murine HSCs exhibiting elevated mitochondrial mass demonstrates enhanced self‐renewal capacity, suggesting functional heterogeneity within the aged HSC pool (Totani et al. 2025). These mitochondria‐enriched HSCs exhibited elevated OXPHOS activity and ATP production without excessive ROS generation, suggesting potential targets for therapeutic intervention. Their study also identified GPR183 as a marker of high‐functioning aged HSCs, linking mitochondrial dynamics directly to stem cell aging and resilience.

Moreover, recent evidence has revealed *DNMT3A*‐mutant HSCs exhibit increased mitochondrial metabolism and one‐carbon flux, providing a clonal fitness advantage under inflammatory stress. Hosseini et al. demonstrated that metformin, by targeting mitochondrial complex I, reverses both the metabolic and epigenetic aberrations in these mutant HSCs, suppressing their clonal expansion in a murine model (Hosseini et al. 2025).

## 4 Aging of the Hematopoietic Niche

### 4.1 Structural and Functional Alterations in the Bone Marrow Microenvironment

The hematopoietic niche in the bone marrow (BM) undergoes substantial age‐related changes that impair its ability to support HSC function. Histologically, aged BM is characterized by reduced vascular density, increased adipogenesis, and a decline in osteoblastic niche cells (Stucker et al. 2020; Ambrosi et al. 2017; Morrison and Scadden 2014). These structural changes alter oxygen gradients and metabolic stress, promote HSC dysfunction, and contribute to myeloid bias.

Age‐associated reductions in classical niche‐derived factors such as CXCL12, IGF‐1, SCF, and G‐CSF have long been recognized as drivers of impaired HSC maintenance and biased differentiation (Nakatani et al. 2023; Pinho and Zhao 2024). Beyond these well‐established mediators, recent work has identified Netrin‐1 as a novel factor whose loss further weakens vascular integrity and exacerbates adipogenesis and DNA damage in the aged niche. The restoration of Netrin‐1, in a murine model, rejuvenates aged niches, restores DNA damage responses (DDR), and improves the capacity of HSCs to self‐renew and reconstitute multilineage hematopoiesis (Ramalingam et al. 2023).

Recent studies have highlighted that not all bone marrow compartments age uniformly. In mice, the calvarial bone marrow shows resistance to several classical hallmarks of niche aging (Koh et al. 2024). Unlike the femoral BM, which exhibits vascular rarefaction, adipogenic replacement, and compromised niche integrity, the skull BM undergoes progressive expansion of marrow space and sinusoidal networks during adulthood with maintained sinusoidal integrity and low adiposity; human calvarial marrow shows analogous features by imaging although mechanistic data are largely murine (Koh et al. 2024). This compartment maintains low adiposity and is less prone to the upregulation of inflammatory cytokines such as IL‐1 and TNF‐α with age, potentially preserving a more supportive environment for hematopoiesis and offering a unique microenvironmental resilience against aging‐induced HSC dysfunction.

Concomitantly, biomechanical changes within the BM niche also contribute to hematopoietic aging. In mice, in situ atomic force microscopy has revealed that BM matrix stiffness increases with age, which negatively regulates the expression of HSC niche factors by bone marrow stromal cells (Zhang, Cao, et al. 2023). Mechanistically, this stiffening activates Yap/Taz signaling, impairing the stromal support of HSCs. Soft matrix conditions, in contrast, promote niche factor expression and facilitate HSC maintenance and lymphopoiesis, suggesting that matrix stiffness represents a previously underappreciated yet targetable dimension of the aging niche.

In humans, aging is also associated with altered spatial relationships between hematopoietic cells and adipocytes within the marrow (Aguilar‐Navarro et al. 2020). Increased BM adiposity correlates with higher density and proximity of CD34^+^ hematopoietic progenitors to adipocytes, potentially promoting myeloid skewing through paracrine or metabolic mechanisms. Despite stable stromal cell numbers, the redistribution of HSPCs toward adipocyte‐rich areas may contribute to the functional decline of hematopoiesis observed in aged individuals. Parallel observations in human femoral bone marrow have shown that increased marrow fat with age is linked to reduced side population HSCs and diminished levels of IGF‐1 and CXCL12, key cytokines involved in HSC maintenance and homing (Tuljapurkar et al. 2011).

### 4.2 Age‐Associated Inflammatory Remodeling of the Hematopoietic Niche

Aging profoundly alters the BM microenvironment. In mice, age‐related deterioration of the niche includes a reduction in osteoprogenitor cells, expansion of pro‐inflammatory LepR^+^ mesenchymal stromal cells (MSCs), and degradation of sinusoidal vasculature, creating a chronically inflamed environment that disrupts HSC quiescence and self‐renewal (Mitchell et al. 2023). Notably, in aged mice, IL‐1β produced by damaged endosteal cells drives inflammation and stress‐induced myelopoiesis in HSPCs, resulting in myeloid‐biased differentiation and impaired regeneration; genetic or pharmacologic blockade of IL‐1 signaling in mice alleviates these defects (Mitchell et al. 2023).

Chronic inflammatory stress is a well‐established driver of HSC dysfunction. Models involving sustained TNF‐α or IFN‐α exposure have shown that prolonged inflammatory signaling induces irreversible HSC proliferation, functional exhaustion, and myeloid skewing, thereby recapitulating features of aged hematopoiesis (Esplin et al. 2011; Pietras et al. 2016; Mitroulis et al. 2018). Building on this, recent murine work has provided definitive evidence that even discrete and temporally separated inflammatory episodes cause long‐lasting and cumulative impairments in HSC self‐renewal capacity, with no recovery observed up to 1 year post‐challenge (Bogeska et al. 2022). This study provides direct proof‐of‐principle that even transient inflammatory insults, when repeated, can program irreversible aging‐like features into the hematopoietic system, including functional exhaustion, myeloid‐skewed differentiation, and reduced clonal complexity.

The gut microbiota also emerges as a key regulator of this inflammatory remodeling. Transplantation of the fecal microbiota from young mice into aged recipients rejuvenates aged HSCs by attenuating inflammatory signaling and restoring lymphoid potential (Zeng et al. 2023). This rejuvenation is associated with reduced IL‐1 signaling, increased expression of *Foxo*‐associated genes, and reshaped metabolite profiles, particularly involving tryptophan derivatives and SCFA‐producing bacteria such as Lachnospiraceae. Recent work using *Dnmt3a*‐mutant mouse models has further revealed that impaired gut barrier integrity with age permits systemic dissemination of the bacterial metabolite ADP‐heptose, which activates ALPK1–NF‐κB signaling in pre‐leukemic HSCs, promoting their clonal expansion and niche inflammation (Agarwal et al. 2025). Collectively, these murine data establish causal links between inflammation, microbiome dysregulation, and HSC aging. Yet, whether similar interventions can modulate human BM niches remains an open question, highlighting an important translational frontier.

### 4.3 Endothelial and Osteoblastic Niche Decline

Bone marrow endothelial cells (BMECs) and osteoblasts are essential components of the hematopoietic niche, supporting vascular integrity and HSC maintenance (Morrison and Scadden 2014; Kopp et al. 2005; Poulos et al. 2015; Taichman and Emerson 1994; Calvi et al. 2003). In mice, aging impairs BMEC function, leading to enhanced vascular permeability, reactive oxygen species (ROS) production, hypoxia, and reduced expression of critical support factors, such as SCF and CXCL12 (Poulos et al. 2017). These changes disrupt angiocrine signaling and skew HSC differentiation toward myeloid lineages (Boueya et al. 2024). Experimental infusion of aged ECs into young mice confirms their detrimental effects on hematopoietic recovery and lineage balance.

In parallel, murine studies have shown that aging reduces osteoblast numbers and function, particularly impairing the support of lymphoid‐biased HSCs (Calvi et al. 2003). Osteoblastic decline exacerbates the shift toward myeloid dominance and diminishes regenerative capacity. Furthermore, the intrinsic composition of HSCs shifts with age, favoring the expansion of myeloid‐biased over lymphoid‐biased clones—an imbalance reinforced by niche deterioration.

Emerging human studies provide correlative evidence for similar processes. Spatial transcriptomic and histological analyses of aged human bone marrow reveal loss of arteriolar endosteal niches, reduced CXCL12^+^ stromal populations, and increased marrow adiposity, each associated with altered HSPC localization and lineage bias (Kovtonyuk et al. 2016; Ho et al. 2019). However, direct functional evidence in humans that endothelial or osteoblastic aging causally impairs HSC support remains limited, with most mechanistic data still derived from murine models.

## 5 Systemic Interactions—Hematopoietic Aging and Other Organs

### 5.1 Hematopoietic Aging and Clonal Hematopoiesis in Systemic Aging

**FIGURE 2:**
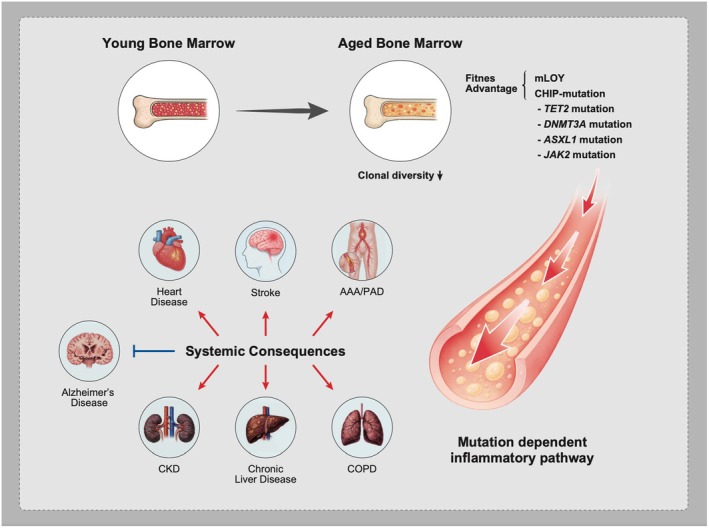
Clonal hematopoiesis as a systemic amplifier of aging‐related disease. With aging, hematopoietic stem cell (HSC) populations progressively lose clonal diversity, while a subset of clones acquires a fitness advantage, leading to clonal dominance. This process is frequently driven by clonal hematopoiesis of indeterminate potential (CHIP)–associated somatic mutations, including *TET2*, *DNMT3A*, *ASXL1*, and *JAK2*, as well as by age‐related chromosomal mosaicism such as mosaic loss of the Y chromosome (mLOY). Expanded mutant HSC‐derived clones promote mutation‐dependent inflammatory programs in circulating myeloid cells, contributing to systemic inflammation and increased risk of age‐related diseases, particularly cardiovascular diseases. *AAA, abdominal aortic aneurysm; PAD, peripheral artery disease; CKD, chronic kidney disease; COPD, chronic obstructive pulmonary disease.

CHIP is associated with a significant increase in all‐cause mortality, primarily driven by cardiovascular events rather than hematologic malignancy. Age‐associated somatic mutations such as *TET2*, *DNMT3A*, and *ASXL1* promote the expansion of mutant hematopoietic clones, driving systemic inflammation and vascular dysfunction (Jaiswal et al. 2017; Jaiswal and Ebert 2019; Cook et al. 2020; Jaiswal and Libby 2020) (Figure 2).

Mechanistic research using murine models has revealed that mutant myeloid cells harboring CHIP‐associated mutations secrete elevated levels of proinflammatory cytokines such as IL‐1β and IL‐6, promoting vascular inflammation and atherogenesis (Fuster et al. 2017; Sano et al. 2018; Fidler et al. 2021; Sato et al. 2024). Consistently, large‐scale human genomic analyses have shown that individuals with CHIP exhibit elevated circulating IL‐6 levels, further supporting the presence of a systemic proinflammatory state in CHIP carriers (Bick et al. 2020). However, not all CHIP‐associated mutations confer identical biological behavior or therapeutic responsiveness. For example, IL‐1β levels are significantly elevated only in *TET2*‐mutant CHIP, whereas IL‐18 is selectively upregulated in *JAK2*‐mutant CHIP, suggesting that distinct driver mutations engage divergent inflammatory pathways (Bick et al. 2020). Consistent with this, a secondary analysis of the CANTOS trial demonstrated that treatment with the IL‐1β‐neutralizing antibody canakinumab significantly reduced recurrent cardiovascular events in individuals harboring *TET2* mutations, but not in those with non*‐TET2* mutations or in CHIP‐negative individuals (Svensson et al. 2022). Analyses of the UK Biobank cohort revealed that CHIP is associated with an increased risk of abdominal aortic aneurysm (AAA) (Tan et al. 2025). This excess risk was attenuated in individuals carrying the IL6R p.Asp358Ala polymorphism, which is known to reduce IL‐6 signaling. Notably, however, among the major CHIP driver mutations, *ASXL1*‐mutant CHIP conferred the highest AAA risk, and this risk was not mitigated by the IL6R p.Asp358Ala variant. These observations suggest that CHIP is not a uniform entity and that its pathogenic and therapeutic implications may be highly mutation‐specific.

CHIP has also been causally linked to multiple forms of CVD, including coronary artery disease (CAD), heart failure (HF), stroke, and peripheral artery disease (PAD) as well as non‐vascular disease like chronic kidney disease, chronic obstructive pulmonary disease, and chronic liver disease (Jaiswal et al. 2017; Dorsheimer et al. 2019; Pascual‐Figal et al. 2021; Yu et al. 2021; Tan et al. 2025; Arends et al. 2023; Büttner et al. 2023). Although CHIP is most often discussed as a deleterious, pre‐malignant state, emerging data indicate that its impact may not be uniformly harmful. For instance, certain mutations, particularly in *TET2*, have been associated with a reduced risk of Alzheimer's disease, possibly via altered microglial activity (Bouzid et al. 2023; Vicario et al. 2025). While recent studies report that the prevalence of CHIP increases notably among long‐lived individuals and have speculated about possible homeostatic benefits under aging‐related stress, these observations remain correlative and do not establish a causal or protective role for CH in longevity (Wang et al. 2024; Mitchell et al. 2022). These findings suggest that CHIP may exert context‐dependent effects, occasionally conferring advantages, even while overall it remains a major age‐related risk factor for hematologic and cardiovascular disease.

In addition to CHIP, other age‐related hematopoietic alterations contribute to cardiovascular pathology. For instance, mosaic chromosomal alterations (mCAs) and mosaic loss of the Y chromosome (mLOY) have been associated with increased cardiovascular risk via mechanisms overlapping with CHIP, such as impaired immune regulation and heightened vascular inflammation (Forsberg et al. 2014; Bruhn‐Olszewska et al. 2025).

Moreover, emerging evidence suggests that systemic inflammatory signals from non‐hematopoietic organs can also influence hematopoietic stem cell behavior. Notably, a murine stroke model revealed that acute cerebral ischemia triggers inflammatory cascades that epigenetically reprogram bone marrow HSCs, inducing sustained myeloid bias and contributing to remote cardiac fibrosis (Simats et al. 2024). This finding highlights a bidirectional axis between injured organs and the hematopoietic system, further emphasizing the central role of hematopoiesis in orchestrating age‐related disease across multiple organ systems.

### 5.2 Parabiosis Studies and Rejuvenation Insights

Heterochronic parabiosis—linking the bloodstreams of young and old animals—has revealed that youthful systemic factors can broadly rejuvenate aged tissues. Old mice paired with young partners exhibit enhanced muscle regeneration, improved hepatic regenerative capacity, revitalized neurogenic activity in the brain, and restored hematopoietic function (Conboy et al. 2005; Conboy et al. 2015). Such systemic rejuvenation has been observed in both mice and rats, spanning multiple organ systems. These benefits extend to mitotically active progenitors (e.g., neural stem/progenitor cells, muscle satellite cells, and HSCs) as well as post‐mitotic cells such as neurons, highlighting that age‐related decline is largely due to an adverse extrinsic milieu rather than irreversible intrinsic damage (Rando and Chang 2012; Conboy et al. 2013; Kase et al. 2019).

Mechanistically, exposure to young blood modulates key signaling pathways associated with aging. For example, elevated Wnt signaling in old muscles skews stem cells toward fibrogenic fates at the expense of regeneration, a change driven by age‐related systemic factors. In a young circulatory environment, this pro‐fibrotic influence is diminished, allowing aged muscle stem cells to resume youthful myogenesis (Brack et al. 2007). Moreover, the rejuvenation effects can persist even after parabiotic pairing, implying that youthful factors induce durable molecular changes—possibly a partial epigenetic reprogramming of aged cells—that reset tissue repair and maintenance programs (Conboy et al. 2005). Furthermore, Ma et al. and Pálovics et al. both performed *single‐cell RNA‐seq atlases across multiple organs* in heterochronic parabiosis, showing that exposure to young circulation partially reverses aging‐associated transcriptional programs in a cell type– and tissue‐specific manner (Ma et al. 2022; Pálovics et al. 2022). Notably, inflammatory and interferon response pathways were attenuated, whereas mitochondrial and proteostasis networks shifted toward more youthful states.

Within the hematopoietic system, heterochronic parabiosis produces mixed results. Although initial studies showed limited rejuvenation of aged hematopoietic stem cells (HSCs) at the functional level, single‐cell transcriptomic analysis has revealed that youthful circulation reverses age‐associated gene expression patterns in subsets of HSCs, including genes involved in cytokine signaling, chromatin remodeling, and circadian regulation (Kuribayashi et al. 2020; Zhang, Lee, et al. 2023). Similarly, bone marrow stromal cells (BMSCs), which support hematopoiesis and bone remodeling, exhibit improved chondrogenesis and ECM production under a youthful systemic influence, contributing to enhanced bone repair (Baht et al. 2015).

Encouraged by murine models, translational research has moved toward human applications. Early‐stage clinical trials, such as those involving human umbilical cord blood plasma (hUCBP), have shown modest benefits on DNA methylation GrimAge and some clinical biomarkers without serious adverse effects (Clement et al. 2022). The “PLASMA” trial and similar studies have tested the safety of young plasma transfusions in older individuals with Alzheimer's or Parkinson's disease (Sha et al. 2019; Parker et al. 2020). However, clear evidence of efficacy remains elusive, and more robust, long‐term randomized trials are required.

The commercialization of youthful blood components raises broader ethical and logistical concerns. The limited supply of young plasma, coupled with the increasing demand among aging populations, risks exacerbating healthcare disparities.

### 5.3 Senescent Cell‐Driven Immune Evasion, Systemic Impact, and Therapeutic Strategies

Aging leads to immune senescence, characterized by diminished immune surveillance, as well as the progressive senescence of somatic cells across multiple organs. Under normal physiological conditions, senescent cells are recognized and cleared by cytotoxic lymphocytes, including CD4^+^ and CD8^+^ T cells, which target stress‐induced or viral antigens such as HCMV‐gB expressed on senescent cells (Hasegawa et al. 2023). However, senescent cells acquire immune evasion mechanisms that enable them to escape clearance by cytotoxic T cells, thereby exacerbating systemic aging.

Senescent cells in various tissues upregulate immune checkpoint molecules such as programmed cell death ligand 1 (PD‐L1) while secreting immunosuppressive SASP factors including IL‐6 and TGF‐β (Onorati et al. 2022). These changes create a local microenvironment that inhibits immune‐mediated clearance. The resultant immune evasion by senescent cells promotes their accumulation, driving chronic low‐grade inflammation, tumorigenesis, and multi‐organ dysfunction (Gorgoulis et al. 2019).

Emerging evidence shows that senescent cells exhibit heterogeneous expression of PD‐L1, which facilitates their evasion of CD8^+^ T cell‐mediated cytotoxicity. With age, PD‐L1^+^ senescent cells accumulate in vivo, exhibiting enhanced SASP‐driven inflammation. Notably, immune checkpoint blockade restores immune‐mediated clearance of PD‐L1^+^ senescent cells, leading to functional improvements such as better lung function, reduced hepatic steatosis, and enhanced motor performance in aged mice (Wang et al. 2022; Wang et al. 2024). These findings highlight the central role of the PD‐1/PD‐L1 pathway in mediating immune escape by senescent cells, linking local cellular senescence to systemic aging.

Therapeutic strategies targeting senescent cells, including senolytics such as dasatinib + quercetin, navitoclax, and fisetin, have demonstrated benefits in multiple aging organs in mice, improving vascular function, bone integrity, metabolic homeostasis, and physical performance, with early pilot studies suggesting feasibility in humans (Clayton et al. 2023; Roos et al. 2016; Farr et al. 2017; Xu et al. 2018; Hickson et al. 2019). Similarly, SGLT2 inhibition has been shown to reduce senescent cell burden and systemic inflammation in aged mice (Katsuumi et al. 2024). These data establish senolysis as a promising anti‐aging strategy; however, whether similar benefits extend to the hematopoietic system is unknown, as no studies to date have demonstrated rejuvenation of aged HSCs or reversal of bone marrow senescence through senolytic treatment. Further work is required to determine whether systemic senolysis can modulate clonal hematopoiesis, immune aging, or age‐associated remodeling of the bone marrow niche, representing an important translational frontier for the field.

### 5.4 Repositioning Hematopoietic Aging as a Systemic Therapeutic Axis

**FIGURE 3:**
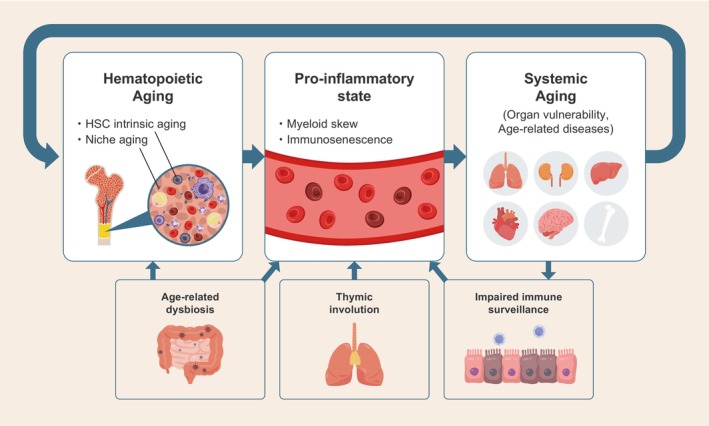
Overview of the bidirectional interactions between hematopoietic aging and systemic aging. Hematopoietic stem cell (HSC) – intrinsic aging and age‐related alterations in the bone marrow niche drive myeloid skewing, immunosenescence, and a chronic pro‐inflammatory state. These hematopoietic changes impair immune surveillance and contribute to systemic aging, increasing organ vulnerability and susceptibility to age‐related diseases. In turn, systemic inflammatory cues and extrinsic age‐related factors, including dysbiosis and thymic involution, further exacerbate hematopoietic dysfunction, establishing a feed‐forward loop.

Hematopoietic aging extends far beyond the confines of the bone marrow, functioning as a central regulator of systemic decline through its influence on inflammation, immune dysregulation, and inter‐organ communication. Moreover, reciprocal signaling from peripheral organs, such as the brain and gut, further shapes hematopoietic aging, highlighting the bidirectional nature of these interactions (Figure 3).

Therapeutically, targeting hematopoietic aging offers a unique opportunity to intercept multiple age‐related diseases through a common upstream axis. Anti‐inflammatory agents such as IL‐1β inhibitors have shown mutation‐specific efficacy in CHIP‐related cardiovascular disease. Senolytics and immune checkpoint blockade may mitigate the pro‐inflammatory burden of senescent cells, thereby restoring immune competence and reducing systemic frailty. Additionally, interventions such as microbiota transplantation, metabolic reprogramming, and parabiosis‐inspired strategies hold promise for rejuvenating hematopoietic and immune function. In the context of clonal hematopoiesis, particularly involving *DNMT3A* mutations, clonal expansion and the associated cardiovascular risk may be modulated through interventions targeting microbial and metabolic pathways.

Recognizing hematopoietic aging as a systemic amplifier of age‐related pathology shifts the paradigm from organ‐specific treatments to integrative, stem cell‐centered approaches. Future research should prioritize precision targeting of hematopoietic alterations, incorporating clonal, inflammatory, and metabolic profiles to guide personalized interventions aimed at extending healthspan and mitigating multimorbidity in the elderly.

## 6 Conclusion

The aging of the hematopoietic system is a hallmark of organismal aging, driven by intrinsic stem cell alterations, niche dysfunction, and systemic inflammatory changes. Age‐related hematopoietic dysfunction manifests as myeloid skewing, reduced lymphopoiesis, and increased susceptibility to infections, malignancies, and inflammatory diseases. Mechanistically, intrinsic alterations such as genomic instability, epigenetic drift, and metabolic reprogramming impair HSC function, while niche aging and systemic factors further intensify these effects. Importantly, hematopoietic aging is increasingly recognized as a central amplifier of systemic aging, influencing cardiovascular health, bone homeostasis, and immune regulation through clonal hematopoiesis and chronic inflammation. This reconceptualization opens new therapeutic possibilities: by targeting intrinsic and extrinsic drivers of hematopoietic aging, including senescent cells, mitochondrial dysfunction, and the aging microbiome, we may rejuvenate the hematopoietic system, mitigate multimorbidity, and extend healthspan. Advancing our understanding of hematopoietic aging is thus critical for designing stem cell‐centered strategies to promote healthy aging in the broader population.

## Author Contributions

The original draft was written by M.M., S.H., and conceptualization was done by Y.K. Editing of the manuscript was done by S.O. and Y.K. Visualization was handled by Y.K. M.M.

## Funding

This research was supported by the Fujita Mind‐Brain Research & Innovation Center for Drug Generation (Fujita Mind‐BRIDGe) of Japan's Peak Research Universities (J‐PEAKS) Program (JPJS00420240019) funded by the Japan Society for the Promotion of Science (JSPS).

## Ethics Statement

The authors have nothing to report.

## Conflicts of Interest

The authors declare no conflicts of interest.

## Data Availability

The authors have nothing to report.
